# γ-Tocotrienol and α-Tocopherol Ether Acetate Enhance Docetaxel Activity in Drug-Resistant Prostate Cancer Cells

**DOI:** 10.3390/molecules25020398

**Published:** 2020-01-18

**Authors:** Spencer Asay, Andrew Graham, Sydney Hollingsworth, Bradley Barnes, Richard V. Oblad, David J. Michaelis, Jason D. Kenealey

**Affiliations:** 1 Department of Nutrition, Dietetics, and Food Science, Brigham Young University, Provo, UT 84602, USA; spencerhasay@gmail.com (S.A.); andrew.graham261@gmail.com (A.G.); sydneyduke1@hotmail.com (S.H.); barnesbradley02@gmail.com (B.B.); richard.oblad@gmail.com (R.V.O.); 2 Department of Chemistry and Biochemistry, Brigham Young University, Provo, UT 84602, USA; david_michaelis@byu.edu

**Keywords:** prostate cancer, vitamin E, mixture design response surface methodology, combination chemotherapy

## Abstract

Prostate cancer is the second most commonly diagnosed cancer in men, and metastatic prostate cancer is currently incurable. Prostate cancer frequently becomes resistant to standard of care treatments, and the administration of chemotherapeutic drugs is often accompanied by toxic side effects. Combination therapy is one tool that can be used to combat therapeutic resistance and drug toxicity. Vitamin E (VE) compounds and analogs have been proposed as potential non-toxic chemotherapeutics. Here we modeled combination therapy using mixture design response surface methodology (MDRSM), a statistical technique designed to optimize mixture compositions, to determine whether combinations of three chemotherapeutic agents: γ-tocotrienol (γ-T3), α-tocopherol ether acetate (α-TEA), and docetaxel (DOC), would prove more effective than docetaxel alone in the treatment of human prostate cancer cells. Response surfaces were generated for cell viability, and the optimal treatment combination for reducing cell viability was calculated. We found that a combination of 20 µM γ-T3, 30 µM α-TEA, and 25 nm DOC was most effective in the treatment of PC-3 cells. We also found that the combination of γ-T3 and α-TEA with DOC decreased the amount of DOC required to reduce cell viability in PC-3 cells and ameliorated therapeutic resistance in DOC-resistant PC-3 cells.

## 1. Introduction

With 174,650 new cases diagnosed in 2018, prostate cancer is the most common cancer in men in the United States [1]. Of those diagnosed with prostate cancer, 10–20% will develop the more aggressive castration-resistant prostate cancer (CRPC) within 4–5 years, and more than 84% of those diagnosed with CRPC have metastatic cancer at the time of diagnosis [2]. Metastatic prostate cancer is currently incurable, with patients living on average only 9–13 months beyond diagnosis [2,3]. While the prevalence of low-risk prostate cancer has declined, metastatic prostate cancer increased by 72% between 2004 and 2013 [4].

Chemoresistance is one of the greatest challenges faced by cancer therapies. Most prostate cancers begin androgen-sensitive and can thus be treated with androgen-deprivation therapy; however, cases of advanced prostate cancer frequently become castration-resistant after 2–3 years of regular treatment [5,6]. Prostate cancer also frequently becomes resistant to common chemotherapeutic drugs [6,7,8]. Further, docetaxel, like many standard chemotherapeutic drugs, is often accompanied by adverse toxic side effects [9,10]. Despite the challenges presented by chemoresistance and drug toxicity, there are promising ways of combating such issues. One such way is by employing a combination of treatments that function through different mechanisms [11].

Mixture-design response surface methodology (MDRSM) is a statistical and mathematical model that can be used to test for optimal mixtures of two or more compounds by generating three-dimensional maps based on interpolation of measured data points. To create a response surface from which unique, effective combinations can be interpolated, one collects a series of data points for defined combination ratios. In the MDRSM model, the researcher has the flexibility to set his or her own parameters around the concentrations of each drug, such as testing between the IC_25_ and the IC_75_ [12]. MDRSM was proposed and shown to be an effective model for optimizing chemotherapeutic treatments by Oblad et al. in 2018 [13].

The Chou-Talalay statistical model can be used to determine whether two compounds interact synergistically, additively, or antagonistically [14]. The Chou-Talalay method is most commonly used to test drug combinations but is limited by the fact that it can only directly analyze combinations of two drugs and is focused on constant ratio combinations. Oblad et al. showed that MDRSM can measure the effectiveness of nonconstant ratio combinations of two or more drugs and predict the ideal combination. With their unique strengths, MDRSM and Chou-Talalay can be used in tandem to help determine the most effective combination of chemotherapeutics to treat advanced, drug-resistant prostate cancer.

Docetaxel (DOC) is a conventional chemotherapeutic drug used to treat advanced prostate cancer [15]. DOC is a first-line drug for metastatic CRPC [16] that works by inhibiting tubulin and preventing progression through mitosis [17]. α-tocopherol ether acetate (α-TEA) is a vitamin E (VE) analog that has been shown to be an effective treatment for prostate cancer both in-vitro and in-vivo [18], and is now in a phase 1 clinical trial for patients with advanced cancer [19]. Findings in the literature indicate that α-TEA acts through a number of cellular and molecular mechanisms to exert its anticancer effects. Specifically, research has shown that α-TEA affects growth and motility of cancer cells through targeting RhoA/ROCK signaling, activates both extrinsic and intrinsic apoptotic pathways, and suppresses antiapoptotic Akt and Erk targets [20,21]. Other findings suggest that α-TEA stimulates the antitumor immune response, induces membrane ceramide accumulation, activates extrinsic death receptors, and causes mitochondrial destabilization [22,23]. α-tocopherol ether butyrate (α-TEB) is an untested VE analog that is structurally similar to α-tocopherol succinate except it cannot be easily hydrolyzed by endogenous esterase activity in cells. γ-tocotrienol (γ-T3) is a naturally occurring VE isomer found in many plant foods [24]. It is found in particularly high quantities in palm oil and rice bran oil. Tocotrienols have antioxidant activity and have been shown to be more potent anti-cancer molecules than the more abundant tocopherol members of the VE family [25]. γ-T3 has been shown to inhibit prostate cancer cell growth and has been shown to enhance DOC-induced apoptosis [26,27]. It has been suggested that γ-T3 exerts it cytotoxic effects through a number of mechanisms including modulation of pro-survival (Id-1, Id-3, EGF-R, and NF-*κ*B) and pro-apoptotic (JNK) pathways, targeting the Ang-1/Tie-2 signaling pathway 2, and modulating eicosanoids and sphingolipids [27,28,29].

In this study, we use MDRSM and Chou-Talalay modeling to identify ideal mixtures of DOC and VE compounds (α-TEA and γ-T3) to treat advanced prostate cancer cells. We chose these two compounds because γ-T3 targets the cancer stem cell population and sensitizes the cells to DOC, and α-TEA targets tumor cells while sparing normal cells [18,30,31]. PC-3 and DU-145 human prostate cancer cells are derived from metastatic prostate cancer and demonstrate chemoresistance to many common chemotherapeutics [32,33]. As such, PC-3 and DU-145 cells provide an excellent representative model for assessing chemoresistant properties in vitro. In our experimental design, we tested 10 defined combination ratios utilizing different concentrations of three anti-cancer compounds: DOC, α-TEA, and γ-T3. We generated response surfaces for cell viability to find the optimal combination for the treatment of PC-3 and DU-145 human prostate cancer cells. Additionally, we developed a model for DOC-resistance by culturing PC-3 cells in increasing concentrations of DOC until cells began to freely grow and proliferate in the presence of DOC.

## 2. Results

### 2.1. IC_50_ Determination

**Figure 1:**
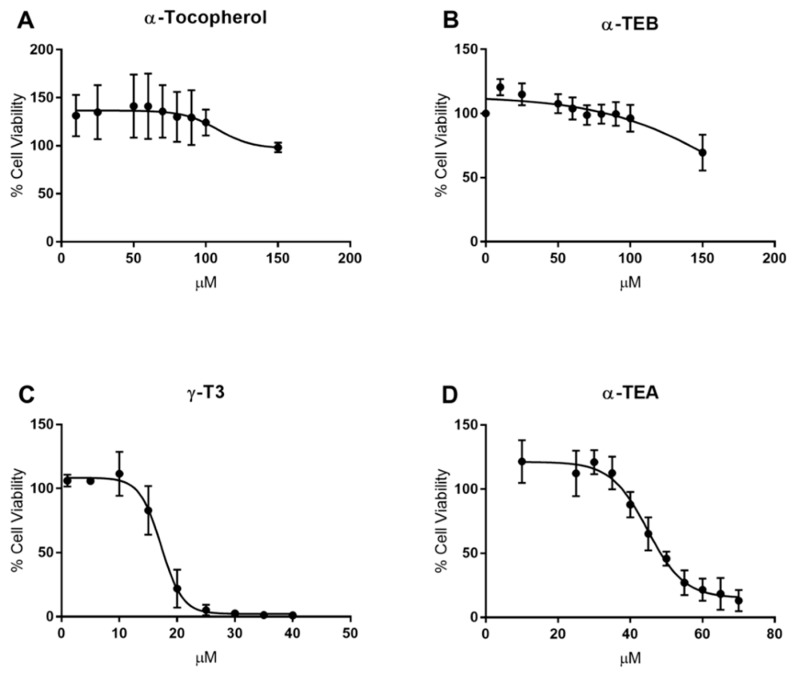
IC_50_ graphs for α-tocopherol (α-TOC), α-tocopherol ether butyrate (α-TEB), γ-tocotrienol (γ-T3), and α-tocopherol ether acetate (α-TEA) in PC-3 human prostate cancer cells. Cell viability at various concentrations over the course of 48 h was assessed for each compound. α-TOC and α-TEB had no effect on cell viability (**A**,**B**). α-TEA and γ-T3 inhibited cell viability in a dose-dependent manner (**C**,**D**). IC_50_ values for these compounds were calculated with a 95% confidence interval: (**C**) γ-T3, 17.0 ± 1.0 µM; (**D**) α-TEA, 45.0 ± 2.3 µM.

To assess the efficacy of α-tocopherol (α-TOC), α-TEB, α-TEA, and γ-T3 against advanced prostate cancer, the IC_50_ of each compound was first determined in PC-3 human prostate cancer cells. In addition, the Chou-Talalay and MDRSM analysis methodologies require that the IC_50_ of each compound of interest be known. Consistent with what has been observed in the literature, treatment with α-TOC elicited virtually no effect on cell viability [34] (Figure 1A). α-TEB also had no effect on cell viability (Figure 1B). In contrast, γ-T3 and α-TEA inhibited cell viability in a dose-dependent manner (Figure 1C,D). IC_50_ values for γ-T3 and α-TEA were calculated with a 95% confidence interval and found to be 17.0 ± 1.0 µM and 45.0 ± 2.3 µM, respectively. These IC_50_ values are similar to those previously measured in human serum and could be achievable in tumors [35,36]. The IC_50_ curves for γ-T3 and α-TEA displayed a near step function drop in cell viability. Treatment with 14 µM γ-T3 resulted in a negligible effect on cell viability, while treatment with 20 µM γ-T3 trended near complete cell death. Similar effects were observed with α-TEA treatment, with the IC_50_ curve decreasing from 100% cell viability to near 0% viability over a 30–60 µM range.

### 2.2. Cell Viability Responses for MDRSM Combinations

**Figure 2:**
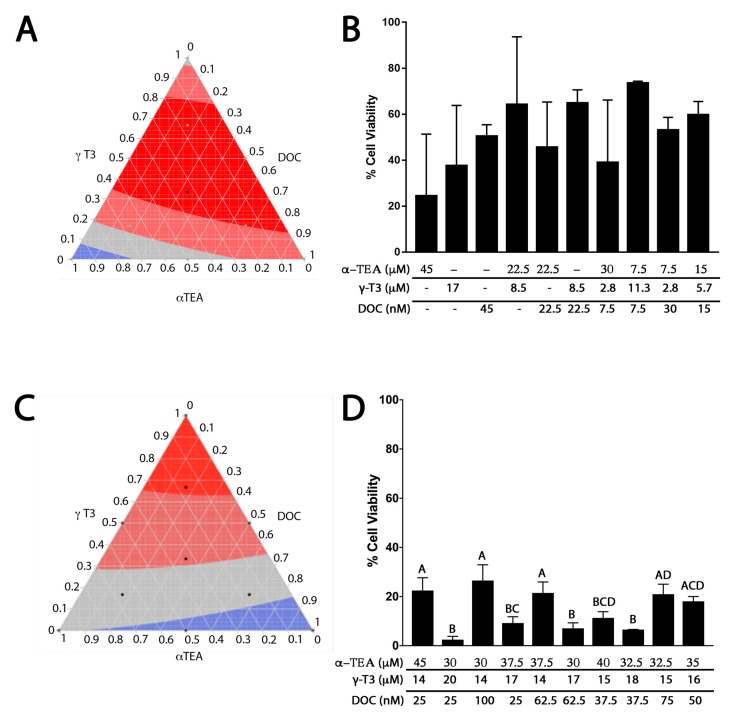
Analysis of γ-T3, α-TEA, and docetaxel (DOC) treatment combinations in PC-3 cells. Black dots on the ternary plots represent points where data were collected to produce the mixture design response surface methodology (MDRSM). Bright red areas represent the most effective combinations for reducing cell viability. Deep red, gray, and blue areas represent decreasing levels of effectiveness in descending order. (**A**) Ternary plot displaying MDRSM analysis conducted with the vertices corresponding to each of the three compounds at their respective IC_50_ values. (**B**) Bar graph displaying cell viability data used to generate ternary plot A. The data were highly variable and only treatment with 45 µM α-TEA was observed to be significantly different from control (*p* = 0.0390). No statistical differences reported among any of the other compounds/mixtures. (**C**) Ternary plot displaying MDRSM analysis conducted with the mixture at each vertex containing the lowest dose observed to have maximal effect for one compound, and the highest concentrations of the other two compounds that elicited no effect (γ-T3 low: 14 µM, high: 20 µM; α-TEA low: 30 µM, high: 45 µM; and DOC low: 25 nM, high: 100 nM). (**D**) Bar graph displaying cell viability data used to generate ternary plot B. The most effective combination for reducing cell viability (combination 2; 97.53% reduction relative to control (*p* < 0.0001) consisted of 30 µM α-TEA, 20 µM γ-T3, and 25 nm DOC, though this result was not significantly different from combinations 4 (90.77%), 6 (92.92%), 7 (88.69%), or 8 (93.42%). Several combinations containing higher concentrations of VE compounds with lower concentrations of DOC were associated with significantly greater reduction in cell viability compared to mixtures containing higher concentrations of DOC with lower concentrations of vitamin E (VE) compounds. Specifically, this result was observed for combinations 6 (*p* < 0.0001), 7 (*p* = 0.0007), and 8 (*p* < 0.0001), which contained 62.5, 37.5, and 37.5 nM DOC respectively, compared to combination 3 (73.47% reduction), which contained 100 nM DOC.

To determine the most effective combination of γ-T3, α-TEA, and DOC for reducing cell viability in PC-3 human prostate cancer cells, a response surface was generated using MDRSM treatment combinations. As shown in Figure 2A, MDRSM was initially employed in the present work with each vertex of the ternary plot defined by each individual compound at its respective IC_50_ value as calculated in Figure 1. The combinations generated using this approach were found to be no more effective than the individual compounds in isolation. Due to there being so little difference between each of the individual points, the MDRSM analysis calculated the optimal concentration (red) to be over half of the MDRSM area. It was speculated that the steepness of the IC_50_ curves for γ-T3 and α-TEA, and the relatively narrow activity ranges of these compounds might account for the lack of any synergistic or additive effect. It was discovered that using the proportions outlined by standard MDRSM protocol resulted in mixtures containing concentrations of each VE compound that were below the lower limits of the activity ranges described in Figure 1. Specifically, the γ-T3 concentrations in the mixtures at 67%, 50%, 33%, and 16% points on the MDRSM were 11 µM, 8.5 µM, 5.6 µM, and 2.8 µM, respectively. For α-TEA, the concentrations at those points were 30 µM, 22.5 µM, 15 µM, and 7.5 µM. All of these said concentrations were below the values at which any effects were observed for γ-T3 or α-TEA in PC-3 cells.

To ensure that no compound was diluted below its range of efficacy, a floor was determined for each compound based on the activity ranges described previously. Using the IC_50_ data described in Figure 1, it was determined that 30 µM, 14 µM, and 25 µM were the highest concentrations of α-TEA, γ-T3, and DOC, respectively, at which no activity was observed. These concentrations were thus taken to be essentially “zero” and became the lowest concentrations of these compounds used in any of the MDRSM combination mixtures. The data in Figure 2D demonstrate that using these ranges produced combinations that yielded substantially less variable results and were more effective in reducing cell viability than those in Figure 2B. The percent differences between the data in Figure 2B,D ranged from 2–67%, yet these differences were all nonsignificant, likely due to the high degree of variability found in the data in Figure 2B. The most effective combination for reducing cell viability in PC-3 cells consisted of 30 µM α-TEA, 20 µM γ-T3, and 25 nm DOC. This and other combinations, as shown above, were found to be significantly more effective than the combination with the highest concentration of DOC, suggesting that DOC is not as effective alone as it is when used in combination with VE analogs.

**Figure 3:**
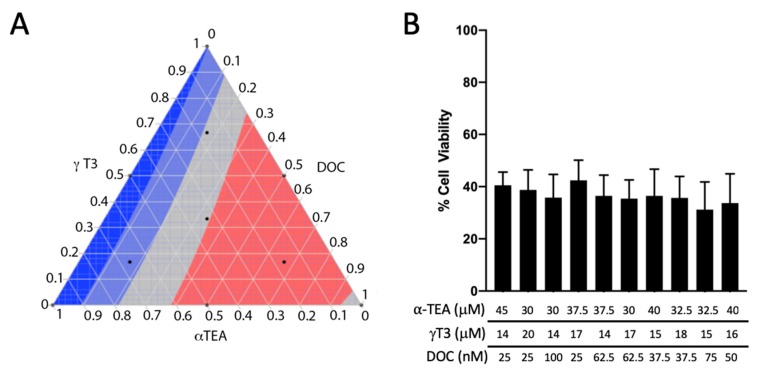
Analysis of γ-T3, α-TEA, and DOC treatment combinations in DU-145 cells. (**A**) Ternary plot displaying MDRSM analysis conducted using the same treatment combinations as used for PC-3 cells in Figure 2C,D. The most effective combination for reducing cell viability was calculated to be 30 µM α-TEA, 16.4 µM γ-T3, and 70 nm DOC. (**B**) Bar graph displaying cell viability data used to generate ternary plot B. No significant differences in treatment response were detected among the various combinations.

To further investigate the effectiveness of combination chemotherapy consisting of γ-T3, α-TEA, and DOC in treating advanced prostate cancer, a response surface was generated for cell viability in DU-145 human prostate cancer cells. For ease of comparison against the data collected on PC-3 cells in Figure 2, and given that the IC_50_ values reported in the literature for each of the three compounds in the DU-145 cell line did not differ significantly from those observed in the PC-3 cell line, γ-T3, α-TEA, and DOC were incorporated in the same ratio combinations as in Figure 2C,D for the treatment of DU-145 cells and generation of a corresponding response surface [37,38] (Figure 3A). Interestingly, although similar dose-dependent effects were observed when treating DU-145 cells with γ-T3 or α-TEA (data not shown) no significant differences in treatment response were observed among the various ratio combinations (Figure 3B). It was thus unsurprising, then, that MDRSM analysis calculated the optimal concentration (red) to occupy a relatively large portion of the response surface area. The optimal combination for reducing cell viability in the DU-145 cell line was calculated to be 30 µM α-TEA, 16.4 µM γ-T3, and 70 nm DOC. The DU-145 cells were not used in subsequent experiments because the mixture of DOC with VE analogs was not any more effective than DOC alone in treating DU-145 cells.

### 2.3. Chou-Talalay Analysis

To assess whether dual combinations of α-TEA, γ-T3, and DOC yielded synergistic, additive, or antagonistic effects, Chou-Talalay analysis was conducted using the alamarBlue cell viability assay. The Chou-Talalay analysis requires nonlinear combinations of 0.25× 0.50×, 1×, 2×, and 4× the IC_50_ for each dual combination of the three compounds. Values close to 1 are considered additive (gray box), values below 1 are synergistic, and values over 1 are antagonistic (red box). Combination of α-TEA or γ-T3 with DOC produced additive effects at each multiple of the IC_50_, while combination of α-TEA with γ-T3 yielded extreme antagonistic effects across all multiples (Table 1). That none of these compounds exhibited synergy does not entail that the mixtures are not worth pursuing; as evidenced by the results in Figure 1D, additive effects can still be used to limit the amount of the toxic DOC used to treat advanced prostate cancer, thus likely limiting toxic side effects associated with the drug.

### 2.4. Cell Viability Response in DOC-Resistant PC-3 Cells

**Figure 4:**
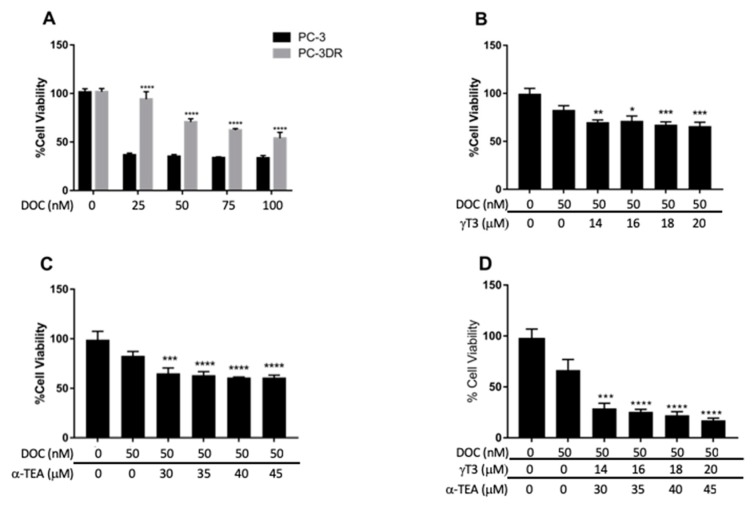
Viability data for DOC-resistant PC-3 (PC-3DR) cells treated using combinations of DOC with α-TEA and/or γ-T3. (**A**) PC-3 and PC-3DR cells treated with 25, 50, 75, and 100 nM DOC. PC-3DR cells exhibited significantly less response to DOC treatment at all concentrations (**** *p* < 0.0001). (**B**) PC-3DR cells treated with 50 nM DOC in combination with 14, 16, 18, and 20 µM γ-T3. Significantly greater reduction in cell viability was observed in all DOC-VE combinations compared to DOC alone (* *p* = 0.0190, ** *p* = 0.0066, *** *p* = 0.0008). (**C**) PC-3DR cells treated with 50 nM DOC in combination with 30, 35, 40, and 45 µM α-TEA. Significantly greater reduction in cell viability was observed in all DOC-VE combinations compared to DOC alone (*** *p* = 0.0002, **** *p* < 0.0001). (**D**) PC-3DR cells treated with 50 nM DOC in combination with 14, 16, 18, and 20 µM γ-T3 and 30, 35, 40, and 45 µM α-TEA. Significantly greater reduction in cell viability was observed in all DOC-VE combinations compared to DOC alone (*** *p* = 0.0001, **** *p* < 0.0001).

To determine the efficacy of γ-T3 and α-TEA in the treatment of DOC-resistant PC-3 cells, DOC-resistant clones were selected for in the process described in the methods section. The alamarBlue cell viability assay was then used to assess cell viability following treatment with the identified compounds in various mixtures. First, to validate that the PC-3DR cells were resistant to DOC, PC-3 and PC-3DR cells were treated with 25, 50, 75, and 100 nM DOC for 48 h (Figure 4A). A significantly reduced response to DOC treatment was observed in the PC-3DR cells at all concentrations. To determine the ability of VE to ameliorate DOC-resistance in prostate cancer cells, PC-3DR cells were treated with mixtures containing DOC and γ-T3 or α-TEA. PC-3DR cells were first treated with 50 nM DOC in combination with 14, 16, 18, and 20 µM γ-T3 for 48 h (Figure 4B). A significantly greater reduction in cell viability was observed for treatment with DOC-VE combinations compared to DOC alone, though no significant differences were observed among the several VE concentrations. The most effective combination consisted of 50 nM DOC and 20 µM γ-T3 and was accompanied by a 17% greater reduction in cell viability relative to 50 nM DOC alone (*p* = 0.0003). As stated, however, this result was not significantly different from those obtained using the other mixture concentrations. PC-3DR cells were then treated with 50 nM DOC in combination with 30, 35, 40, and 45 µM α-TEA for 48 h (Figure 4C). Again, a significantly larger response was observed for treatment with DOC-VE combinations compared to DOC alone, though no significant variance was observed among the different VE concentrations. The combinations containing 50 nM DOC with 40 µM α-TEA and 50 nM DOC with 45 µM α-TEA were equally as effective, with both accompanied by a 22% greater reduction in cell viability relative to 50 nM DOC alone (*p* < 0.0001). Again, this result was not significantly different from those obtained using the other mixture concentrations. Finally, PC-3DR cells were treated with 50 nM DOC in combination with both 14, 16, 18, and 20 µM γ-T3 and 30, 35, 40, and 45 µM α-TEA for 48 h (Figure 4D). Similar results were observed as treatment with the DOC-VE mixtures elicited a significantly greater reduction in cell viability compared to DOC alone, albeit again with no significant differences among the various VE concentrations. The most effective combination consisted of 50 nM DOC, 20 µM γ-T3, and 45 µM α-TEA and was accompanied by a 49% greater reduction in cell viability relative to 50 nM DOC alone (*p* < 0.0001). Again, this result was not significantly different from those obtained using the other concentration mixtures.

Further statistical analysis of the data in Figure 4B–D revealed that treatment with 50 nM DOC combined with both VE compounds was associated with a significantly greater response compared to treatment with 50 nM DOC with an equal dosage of either VE compound. For instance, treatment with a mixture consisting of 50 nM DOC, 20 µM γ-T3, and 45 µM α-TEA saw a 49% greater reduction in cell viability compared to treatment with a mixture containing 50 nM DOC and 20 µM γ-T3 (*p* < 0.0001), and a 44% greater reduction compared to treatment with a mixture containing 50 nM DOC and 45 μM α-TEA (*p* < 0.0001).

## 3. Discussion

Combining standard chemotherapeutics with natural products could decrease toxic side effects, decrease chemotherapeutic resistance, and increase treatment efficacy. Chemotherapeutic compounds in combination target multiple cell death pathways simultaneously, increasing the efficacy over using single agents. Combination chemotherapy has been used for years; however, that the administration of standard chemotherapeutic drugs is often accompanied by debilitating toxic side effects remains a noteworthy obstacle in cancer treatment. Many natural compounds, including VE, have been shown to induce tumor cell death without affecting normal cells. Identifying the most effective mixtures of natural and standard chemotherapeutics can be time-consuming; therefore, we have applied two mixture modeling methods to identify the ideal combinations of DOC, γ-T3, and α-TEA.

Our findings support the notion that VE compounds can reduce the amount of the toxic DOC needed to eliminate advanced prostate cancer cells, and that MDRSM can be used to identify ideal mixtures of chemotherapeutic compounds. Here we have shown that a combination of γ-T3 and α-TEA with DOC significantly reduced the levels of DOC required to decrease tumor cell viability. MDRSM analysis demonstrated that mixtures with concentrations of γ-T3 near the IC_50_ of γ-T3 were most effective in treating PC-3 cells. Conversely, treatment with the same VE mixtures on DU-145 cells elicited no statistically significant effect among the various ratio combinations. Previous studies have shown that DU145 cells are more resistant to cell death than PC-3 cells because the cells have unique redox environments [39]. Due to the negative results obtained using DU-145 cells, further testing using additional prostate cancer cell lines is required to accurately appraise the combinatorial effectiveness of these compounds. This differential effect in PC-3 and DU-145 cells could be exploited in future experiments to determine the molecular mechanism of γ-T3 and α-TEA DOC sensitization. The results obtained from the in vitro assays performed in the present study do not guarantee translational potential. Further testing using animal models is advisable. Further, we demonstrated that DOC-resistance in prostate cancer cells can be at least partially ameliorated through treatment with γ-T3 and α-TEA, as these compounds enhanced the effectiveness of DOC in the treatment of DOC-resistant PC-3 cells.

The Chou-Talalay model showed that combining γ-T3 and α-TEA yielded antagonistic interactions, indicating that these two compounds should not be combined for chemotherapeutic treatment. This effect is likely due to α-TEA-induced degradation of γ-T3. Elevated α-VE intake has been found to reduce plasma concentrations of the more protective γ-VE isoforms, possibly through upregulating metabolic enzymes with high affinity for γ-VE or competitively reducing the export of γ-VE from the liver (the former being more applicable to the in vitro experiments conducted in the present study) [40]. Antagonism between α-VE and γ-VE due to α-VE-induced degradation of γ-VE offers possible explanation for the seemingly contradictory results of the SELECT trial, in which supplementation with α-TOC was associated with an increased risk of prostate cancer [41,42]. This apparently contradictory observation could be due to the fact that the Chou-Talalay model can feasibly only test two compounds at once, thus not accounting for the potential effects of a third compound (DOC) on the nature of the interactions between γ-T3 and α-TEA. This observation could also be due to the molecular changes that occur in the PC-3 cells as they become DOC-resistant. Interestingly, the PC-3DR cells were more sensitive to a combination of the γ-T3 and α-TEA than either of the compounds alone, indicating that, as the tumor changes, so may its ability to metabolize or efflux VE compounds.

Previous studies investigating the chemotherapeutic potential of VE have found the treatment of prostate cancer cells with α-TOC to be virtually ineffectual [34]. Our results corroborate these findings and also evidence that treatment with α-TEB is similarly ineffective. We suspect that α-TEA is effective and α-TOC is not because the acetate moiety is required initially for absorption. Subsequently, the acetate is hydrolyzed off the α-TEA by endogenous esterase activity, which perhaps enables it to then bind to its chemotherapeutic cellular target. α-TEB is presumably not active because, while it may be absorbed, it contains an ether bond that will not be cleaved once it enters the cell, and therefore with not be able to bind to its chemotherapeutic target.

Our results also support observations in the literature that γ-T3 is among the most potent of the naturally occurring VE isoforms in terms of chemotherapeutic efficacy [25]. Previous studies have shown γ-T3 to be effective in reducing cell viability and ameliorating DOC resistance in several prostate cancer cell lines [26,27,30]. In the present study, while treatment of PC-3DR cells with γ-T3 or α-TEA in combination with DOC significantly reduced cell viability relative to treatment with DOC alone, the efficacy of both forms of VE appeared to be blunted, suggesting possible resistance to these compounds as well. Further, while the differences in reduction of cell viability after treatment with DOC in combination with various concentrations of either γ-T3 or α-TEA and treatment with DOC alone were statistically significant, the percent differences themselves were not particularly substantial, calling into question the possible practical significance of this result. When we combined both γ-T3 and α-TEA in the same mixture with DOC, however, we observed a near 50% decrease in cell viability relative to DOC alone, indicating that a mixture of all three compounds could be much more effective than DOC alone in treating advanced, drug-resistant prostate cancer. Further, as noted in the results section above, treatment with DOC in combination with both VE compounds was significantly more effective than DOC in combination with either of VE compounds alone.

Our investigations highlight various limitations of MDRSM for biological work. For example, a compound with a steep IC_50_ curve will have several points that will be well below its effective dose. With that in mind, we determined that the weak effect of combinations in Figure 1A was likely due to these small windows of effectiveness. As outlined in the Results section, the concentration of γ-T3 and α-TEA in almost all combinations is below a concentration that will reduce cell viability based on our IC_50_ calculations. We determined that using specific ranges for each combination would alleviate this issue. It may be that using specific treatment ranges rather than beginning with “zero” is advisable for the application of MDRSM in biological settings. We found using such ranges was best practice in the present study, especially given the unique properties of the compounds of interest. Additionally, MDRSM, when utilized in engineering fields, can lead to exact and consistent results. The variability inherent to the biological sciences presents challenges in obtaining useful models. In this study, we used an in vitro model of PC-3 and DU-145 cells; however, cell-based studies do not demonstrate the effect of the mixtures on normal tissue or how the mixtures are metabolized. Subsequent studies can use MDRSM in animal prostate cancer models to identify mixtures that maximize the effect on tumor cells while minimizing the toxicity to normal tissue.

In conclusion, γ-T3 and α-TEA are more active than α-TOC and α-TEB in advanced prostate cancer cells. γ-T3 and α-TEA can be used to reduce the amount of DOC required to eliminate prostate cancer cells. γ-T3 and α-TEA improve the effectiveness of DOC in reducing cell viability in resistant cells. While our results demonstrate the beneficial effect of combination chemotherapy utilizing γ-T3, α-TEA, and DOC in PC-3 cells, we did not observe the same result in DU-145 cells.

## 4. Materials and Methods

### 4.1. Cell Lines

PC-3 and DU-145 human prostate cancer cells were acquired from ATCC (Rockville, MD, USA) and cultured in F12-K media (PC-3) purchased from Corning Incorporated (Oneonta, NY, USA) or Eagle’s Minimum Essential Medium (DU-145) purchased from ATCC. Media was supplemented with 10% fetal bovine serum and 1% antibiotic (streptomycin/penicillin). Cells were grown in incubator under standard conditions of 37 °C and 5% CO_2_ through 10–25 passages.

### 4.2. Selection of Docetaxel-Resistant Clones

PC-3 cells were initially cultured in 1 ng/mL DOC medium and maintained until DOC-sensitive clones were eliminated. The surviving PC-3 cells repopulated the flask and continued to divide through multiple passages. The same process was repeated using DOC concentrations of 5 ng/mL and finally 10 ng/mL. Once PC-3 cells were freely dividing in 10 ng/mL DOC medium, they were considered resistant and labeled PC-3DR.

### 4.3. Reagents

**Scheme 1:**
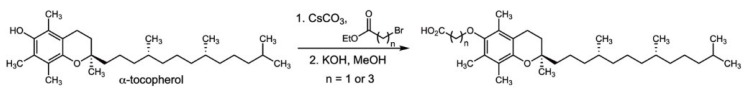
Synthesis of α-TEA and α-TEB.

α-TEA and α-TEB were synthesized at Brigham Young University by Dr. David Michaelis in the Department of Chemistry and Biochemistry (Scheme 1). Pure product was dissolved in dimethyl sulfoxide (DMSO) and stored as a 10 mM stock. γ-T3 was purchased from Cayman Chemical (Ann Arbor, MI, USA) and dissolved in DMSO to be stored as a 10 mM stock. Exposure to light was avoided for vitamin E forms. Docetaxel was obtained from Sigma Aldrich (St. Louis, MO, USA) and dissolved in DMSO to form a 10 uM stock. α-tocopherol (α-TOC) was purchased from Sigma Aldrich (St. Louis, MO, USA) Treatments were prepared by diluting the stock concentrations in F12-K media, with DMSO concentration never above 2.5%. The limited solubility of the compounds required that this relatively high amount of DMSO (2.5%) be added to keep them dissolved in solution. Though higher than what is typically seen in cell-based studies, testing revealed that this concentration of DMSO did not have any discernible effect on mitochondrial functioning or cell viability.

### 4.4. Cell Viability

Cell viability was assessed using the alamarBlue cell viability assay. Cells were grown near confluency, trypsinized, and 5000–10,000 cells were seeded on 96-well plates. Cells were allowed to adhere to the plate for 24 h before treatment with the vehicle control (DMSO 2.5%) or 1 of 10 drug combinations and allowed to incubate for 48 h (37 °C and 5% CO_2_). DMSO only control was set as 100% viability. Each data point was normalized to the DMSO control and presented as a percent reduction in cell viability so that the strongest reduction in cell viability would give us a maximum on the surface. Each combination treatment also had its own control where treatment medium, without cells, was analyzed with alamarBlue to eliminate background fluorescence and interactions between the drugs and the alamarBlue reagent. After 48 h, the cells were treated with 10 μL of alamarBlue (1:10 ratio of alamarBlue to media), and incubated for 6 h (37 °C and 5% CO_2_). The fluorescence was then analyzed on a BMG LABTECH FLUOstar OPTIMA plate reader (BMG LABTECH Inc., Cary, NC, USA) using the 544 nm excitation filter and the 612-emission filter. We ran three plates, with each plate having three samples for each concentration. The concentration of DMSO in all experiments did not exceed 2.5%.

### 4.5. IC_50_ Calculation

The statistical software GraphPad Prism 7 (Graphpad Software, San Diego, CA, USA) was used to calculate the IC_50_ for each compound utilizing the data from the cell viability procedure outlined above. Data was collected for varying concentrations above and below the IC_50_, after which a variable slope non-linear regression model was fit to each compound’s normalized data set (r^2^ > 0.95). The concentration which led to a 50% reduction in cell viability was calculated with a 95% confidence interval. All IC_50_ values utilized fall within this confidence interval.

### 4.6. Mixture Design and Response Surfaces

Ternary plots were generated and analyzed using JMP Pro 13 software (SAS Institute, Cary, NC, USA). Each mixture combination was done in triplicate. Methods followed procedure outlined in paper by Oblad et al., 2018.

The points on the ternary plot represent various combinations with different ratios of each compound. For our experiments, we created custom ranges for our compounds based on the IC_50_ data as determined by the alamarBlue cell viability assay. The ranges were set as such due to some of the compounds being found to have narrow ranges of effectiveness. For example, in our cell viability assay, γ-T3 had no effect until approximately 14 µM, but reduced cell viability by nearly 50% at 17 µM. Had we set the minimum of each compound at 0 and used the range 0–17 µM for γ-T3, we would have expected to see no effect from γ-T3 in the tested combinations until it was at the maximum concentration. However, DOC has an effect on cell viability even at 1–5 nM, and so its minimum concentration was set at 0 nM.

We tested 10 different combinations, and these 10 points create an even spread over the plot that produces a strong surface for differentiating mixtures (Dejaegher and Heyden, 2011). It is then possible to identify the optimal combination from the model. The predicted points defined by the model are given 95% confidence intervals and allow for statistical conclusions to be made about them.

### 4.7. Chou-Talalay Analysis

Cell viability assays (alamarBlue) for 0.25×, 0.50×, 1×, 2×, and 4× IC_50_ for each dual combination of the three compounds were performed and normalized to a 2.0% DMSO control. The data were input into the program CompuSyn (ComboSyn Inc., Paramus, NJ, USA), as well as the concentration ratios, to find the combination index for each combination to determine synergism or antagonism. All data were collected and analyzed as directed by the Chou-Talalay method (Chou and Talalay, 1983).

## Figures and Tables

**Table 1:** Chou-Talalay combination index (CI) chart for dual combinations of γ-T3, α-TEA, and DOC.

	0.25 × IC_50_	0.5 × IC_50_	1 × IC_50_	2 × IC_50_	4 × IC_50_
γ-T3/α-TEA	25,798.9	28,716.9	31,965.0	34,381.7	36,211.6
α-TEA/DOC	1.09516	1.05744	1.02102	0.99697	0.98021
γ-T3/DOC	0.94101	0.93797	0.93494	0.93288	0.93142

Red represents antagonistic CIs, and gray represents additive CIs. CIs were obtained via alamarBlue cell viability assay with three biologic replicates in triplicate.

## References

[1] Siegel R.L., Miller K.D., Jemal A. (2019). Cancer statistics, 2019. CA Cancer J. Clin..

[2] Kirby M., Hirst C., Crawford E.D. (2011). Characterising the castration-resistant prostate cancer population: A systematic review. Int. J. Clin. Pract..

[3] Gundem G., Van Loo P., Kremeyer B., Alexandrov L.B., Tubio J.M.C., Papaemmanuil E., Brewer D.S., Kallio H.M.L., Hognas G., Annala M. (2015). The evolutionary history of lethal metastatic prostate cancer. Nature.

[4] Weiner A.B., Matulewicz R.S., Eggener S.E., Schaeffer E.M. (2016). Increasing incidence of metastatic prostate cancer in the United States (2004–2013). Prostate Cancer Prostatic Dis..

[5] Singer E.A., Golijanin D.J., Miyamoto H., Messing E.M. (2008). Androgen deprivation therapy for prostate cancer. Expert Opin. Pharmacol..

[6] Zhang T., Zhu J., George D.J., Armstrong A.J. (2015). Enzalutamide versus abiraterone acetate for the treatment of men with metastatic castration-resistant prostate cancer. Expert Opin. Pharmacol..

[7] Liang X.J., Meng H., Wang Y., He H., Meng J., Lu J., Wang P.C., Zhao Y., Gao X., Sun B. (2010). Metallofullerene nanoparticles circumvent tumor resistance to cisplatin by reactivating endocytosis. Proc. Natl. Acad. Sci. USA.

[8] Theyer G., Schirmbock M., Thalhammer T., Sherwood E.R., Baumgartner G., Hamilton G. (1993). Role of the MDR-1-encoded multiple drug resistance phenotype in prostate cancer cell lines. J. Urol..

[9] Ho M.Y., Mackey J.R. (2014). Presentation and management of docetaxel-related adverse effects in patients with breast cancer. Cancer Manag. Res..

[10] Baker J., Ajani J., Scotté F., Winther D., Martin M., Aapro M.S., von Minckwitz G. (2009). Docetaxel-related side effects and their management. Eur. J. Oncol. Nurs..

[11] Zimmermann G.R., Lehar J., Keith C.T. (2007). Multi-target therapeutics: When the whole is greater than the sum of the parts. Drug Discov. Today.

[12] Dejaegher B., Heyden Y.V. (2011). Experimental designs and their recent advances in set-up, data interpretation, and analytical applications. J. Pharm. Biomed. Anal..

[13] Oblad R., Doughty H., Lawson J., Christensen M., Kenealey J. (2018). Application of Mixture Design Response Surface Methodology for Combination Chemotherapy in PC-3 Human Prostate Cancer Cells. Mol. Pharmacol..

[14] Chou T.C. (2010). Drug combination studies and their synergy quantification using the Chou-Talalay method. Cancer Res..

[15] Tannock I.F., de Wit R., Berry W.R., Horti J., Pluzanska A., Chi K.N., Oudard S., Theodore C., James N.D., Turesson I. (2004). Docetaxel plus prednisone or mitoxantrone plus prednisone for advanced prostate cancer. N. Engl. J. Med..

[16] Galsky M.D., Dritselis A., Kirkpatrick P., Oh W.K. (2010). Cabazitaxel. Nat. Rev. Drug Discov..

[17] Jordan M.A., Wilson L. (2004). Microtubules as a target for anticancer drugs. Nat. Rev. Cancer.

[18] Jia L., Yu W., Wang P., Sanders B.G., Kline K. (2008). In vivo and in vitro studies of anticancer actions of alpha-TEA for human prostate cancer cells. Prostate.

[19] Curti B.D., Akporiaye E., Sutcliffe K., Bahjat K.S., Koguchi Y., Cramer J., Urba W. (2015). Phase I study of alpha-tocopherlyoxyacetic acid in patients with advanced cancer. J. Immunother. Cancer.

[20] Yao J., Gao P., Xu Y., Li Z. (2016). alpha-TEA inhibits the growth and motility of human colon cancer cells via targeting RhoA/ROCK signaling. Mol. Med. Rep..

[21] Yu W., Shun M.C., Anderson K., Chen H., Sanders B.G., Kline K. (2006). α-TEA inhibits survival and enhances death pathways in cisplatin sensitive and resistant human ovarian cancer cells. Apoptosis.

[22] Hahn T., Jagadish B., Mash E.A., Garrison K., Akporiaye E.T. (2011). α-Tocopheryloxyacetic acid: A novel chemotherapeutic that stimulates the antitumor immune response. Breast Cancer Res..

[23] Li J., Yu W., Tiwary R., Park S.K., Xiong A., Sanders B.G., Kline K. (2010). α-TEA-induced death receptor dependent apoptosis involves activation of acid sphingomyelinase and elevated ceramide-enriched cell surface membranes. Cancer Cell Int..

[24] Ahsan H., Ahad A., Siddiqui W.A. (2015). A review of characterization of tocotrienols from plant oils and foods. J. Chem. Biol..

[25] Kanchi M.M., Shanmugam M.K., Rane G., Sethi G., Kumar A.P. (2017). Tocotrienols: The unsaturated sidekick shifting new paradigms in vitamin E therapeutics. Drug Discov. Today.

[26] Conte C., Floridi A., Aisa C., Piroddi M., Floridi A., Galli F. (2004). Gamma-tocotrienol metabolism and antiproliferative effect in prostate cancer cells. Ann. N. Y. Acad. Sci..

[27] Yap W.N., Chang P.N., Han H.Y., Lee D.T., Ling M.T., Wong Y.C., Yap Y.L. (2008). Gamma-tocotrienol suppresses prostate cancer cell proliferation and invasion through multiple-signalling pathways. Br. J. Cancer.

[28] Jiang Q. (2019). Natural forms of vitamin E and metabolites-regulation of cancer cell death and underlying mechanisms. IUBMB Life.

[29] Tang K.D., Liu J., Russell P.J., Clements J.A., Ling M.-T. (2019). Gamma-Tocotrienol Induces Apoptosis in Prostate Cancer Cells by Targeting the Ang-1/Tie-2 Signalling Pathway. Int. J. Mol. Sci..

[30] Luk S.U., Yap W.N., Chiu Y.T., Lee D.T., Ma S., Lee T.K., Vasireddy R.S., Wong Y.C., Ching Y.P., Nelson C. (2011). Gamma-tocotrienol as an effective agent in targeting prostate cancer stem cell-like population. Int. J. Cancer.

[31] Anderson K., Simmons-Menchaca M., Lawson K.A., Atkinson J., Sanders B.G., Kline K. (2004). Differential response of human ovarian cancer cells to induction of apoptosis by vitamin E Succinate and vitamin E analogue, alpha-TEA. Cancer Res..

[32] Tilley W.D., Bentel J.M., Aspinall J.O., Hall R.E., Horsfall D.J. (1995). Evidence for a novel mechanism of androgen resistance in the human prostate cancer cell line, PC-3. Steroids.

[33] Zhang Y., Wang Y., Yuan J., Qin W., Liu F., Wang F., Zhang G., Yang X. (2012). Toll-like receptor 4 ligation confers chemoresistance to docetaxel on PC-3 human prostate cancer cells. Cell Biol. Toxicol..

[34] Campbell S.E., Stone W.L., Lee S., Whaley S., Yang H., Qui M., Goforth P., Sherman D., McHaffie D., Krishnan K. (2006). Comparative effects of RRR-alpha- and RRR-gamma-tocopherol on proliferation and apoptosis in human colon cancer cell lines. BMC Cancer.

[35] Lippman S.M., Klein E.A., Goodman P.J., Lucia M.S., Thompson I.M., Ford L.G., Parnes H.L., Minasian L.M., Gaziano J.M., Hartline J.A. (2009). Effect of selenium and vitamin E on risk of prostate cancer and other cancers: The Selenium and Vitamin E Cancer Prevention Trial (SELECT). JAMA.

[36] Meganathan P., Jabir R.S., Fuang H.G., Bhoo-Pathy N., Choudhury R.B., Taib N.A., Nesaretnam K., Chik Z. (2015). A new formulation of Gamma Delta Tocotrienol has superior bioavailability compared to existing Tocotrienol-Rich Fraction in healthy human subjects. Sci. Rep..

[37] Yuan B., Liu Y., Yu X., Yin L., Peng Y., Gao Y., Zhu Q., Cao T., Yang Y., Fan X. (2018). FOXM1 contributes to taxane resistance by regulating UHRF1-controlled cancer cell stemness. Cell Death Dis..

[38] McAnally J.A., Gupta J., Sodhani S., Bravo L., Mo H. (2007). Tocotrienols potentiate lovastatin-mediated growth suppression in vitro and in vivo. Exp. Biol. Med..

[39] Jayakumar S., Kunwar A., Sandur S.K., Pandey B.N., Chaubey R.C. (2014). Differential response of DU145 and PC3 prostate cancer cells to ionizing radiation: Role of reactive oxygen species, GSH and Nrf2 in radiosensitivity. BBA Gen. Subj..

[40] Wolf G. (2006). How an increased intake of alpha-tocopherol can suppress the bioavailability of gamma-tocopherol. Nutr. Rev..

[41] Klein E.A., Thompson I.M., Tangen C.M., Crowley J.J., Lucia M.S., Goodman P.J., Minasian L.M., Ford L.G., Parnes H.L., Gaziano J.M. (2011). Vitamin E and the risk of prostate cancer: The Selenium and Vitamin E Cancer Prevention Trial (SELECT). JAMA.

[42] Yang C.S., Li G., Yang Z., Guan F., Chen A., Ju J. (2013). Cancer prevention by tocopherols and tea polyphenols. Cancer Lett..

